# Adaptation through chromosomal inversions in *Anopheles*

**DOI:** 10.3389/fgene.2014.00129

**Published:** 2014-05-21

**Authors:** Diego Ayala, Anna Ullastres, Josefa González

**Affiliations:** ^1^UMR 224 MIVEGEC/BEES, IRDMontpellier, France; ^2^Unité d'Entomologie Médicale, Centre International de Recherches Médicales de FrancevilleFranceville, Gabon; ^3^Comparative and Computational Genomics, Institute of Evolutionary Biology (CSIC-Universitat Pompeu Fabra)Barcelona, Spain

**Keywords:** local adaptation, phenotypic traits, clinal patterns, insecticide resistance, behavioral traits

## Abstract

Chromosomal inversions have been repeatedly involved in local adaptation in a large number of animals and plants. The ecological and behavioral plasticity of *Anopheles* species—human malaria vectors—is mirrored by high amounts of polymorphic inversions. The adaptive significance of chromosomal inversions has been consistently attested by strong and significant correlations between their frequencies and a number of phenotypic traits. Here, we provide an extensive literature review of the different adaptive traits associated with chromosomal inversions in the genus *Anopheles*. Traits having important consequences for the success of present and future vector control measures, such as insecticide resistance and behavioral changes, are discussed.

## Introduction

The ecological success of a species depends on its ability to face the challenges of new biotic and abiotic scenarios. Evolutionary forces such as selection and migration shape the adaptive process (Lenormand, 2002), and the species' genome reflects this evolutionary process through modifications in its sequence and architecture. Among the most prominent adaptation mechanisms are chromosomal inversions (Dobzhansky, 1937; Krimbas and Powell, 1992; Hoffmann et al., 2004), which occur across a large number of taxa, including plants, mammals, fungi, and insects (Hoffmann and Rieseberg, 2008). Inversions join two evolutionary characteristics making them one of the most effective instruments for local adaptation: they involve several or even hundreds of genes, and recombination is drastically reduced in the heterozygote state (Stump et al., 2007; Kulathinal et al., 2009). Together, these characteristics produce a fertile scenario for the spread of genes involved in local adaptation in natural populations (Kirkpatrick and Barton, 2006; Feder and Nosil, 2009; Feder et al., 2011). Inversions can also affect fitness by influencing the expression and/or structure of genes located near their breakpoints (Pérez-Ortín et al., 2002; Puig et al., 2004; Calvete et al., 2012). Besides adaptation, inversions have also been implicated in speciation and sex chromosome evolution (Feder et al., 2003; van Doorn and Kirkpatrick, 2007; Hoffmann and Rieseberg, 2008). Historically, species of the genus *Anopheles* have received a great deal of attention in the study of chromosomal inversions.

**Figure 1:**
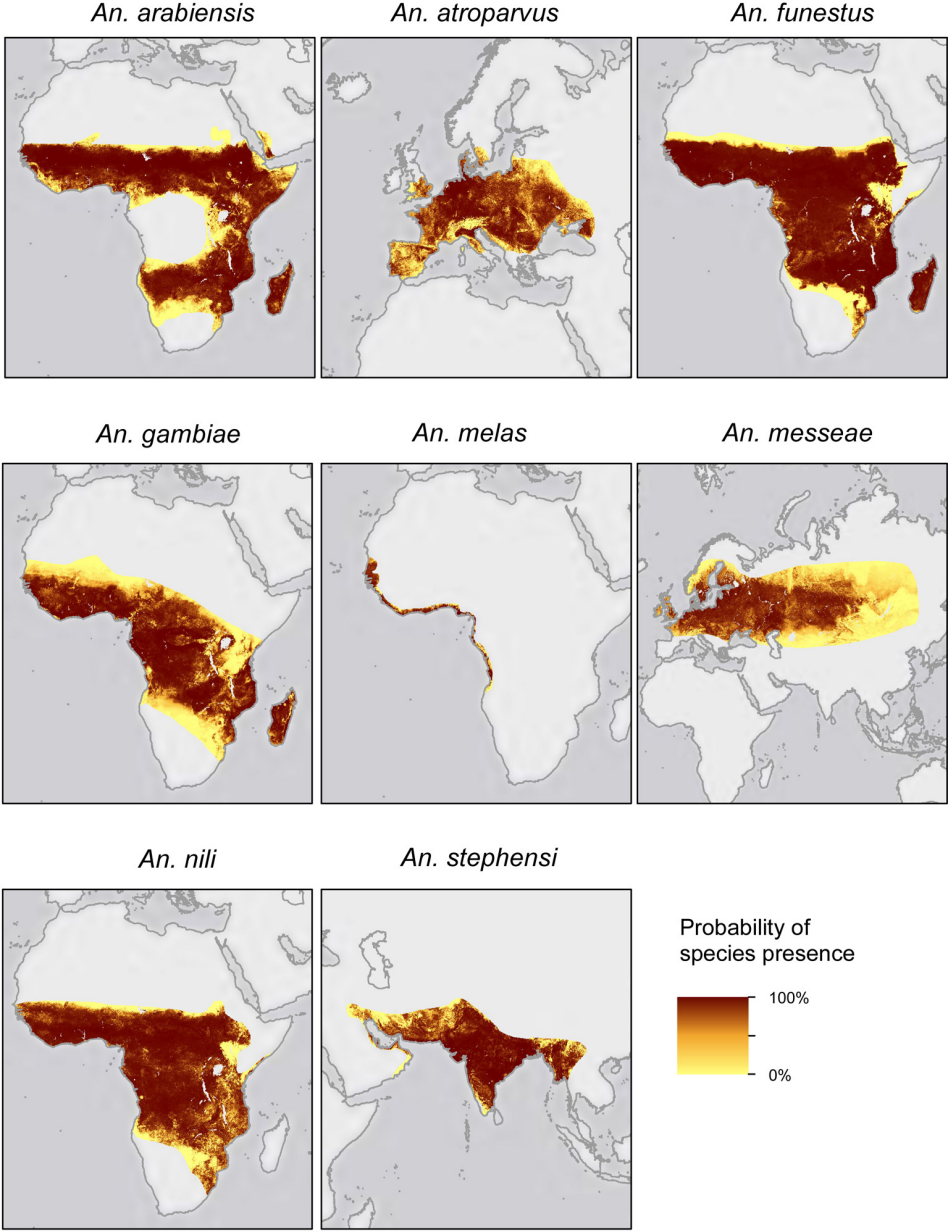
**Geographical distribution of the eight *Anopheles* species reviewed in this work (modified from Sinka et al., 2010)**.

Worldwide interest in the genus *Anopheles* stems from its inauspicious role in the transmission of malaria, responsible for over 1 million deaths per year (WHO, 2012). The success of *Anopheles* species in transmitting malaria parasites is closely related to its ecological capabilities. For instance, the ability of *An. gambiae* to colonize a wide range of ecological settings across the whole of Africa has greatly contributed to making it the world's most proficient malaria vector (Figure 1) (White et al., 2011). Many *Anopheles* species have been extensively studied to better understand their biology, mainly with the aim of implementing vector control strategies. After the success and rapid spread of cytogenetic studies of *Drosophila* (Sturtevant and Dobzhansky, 1936; Krimbas and Powell, 1992), many other diptera species, among them *Anopheles*, were reexamined. Studies of the chromosome architecture in mosquitoes soon followed, as polytene chromosomes were readily accessible from different tissues, in particular nurse cells of half-gravid females and larvae salivary glands (Della Torre, 1997). Before long, natural populations of many important mosquito vectors were the subject of cytogenetic studies.

One of the most fertile fields of cytogenetic research in *Anopheles* is the study of inversions as speciation markers. Fixed inversions among taxonomically identical populations has led to the identification of species complexes (Green and Hunt, 1980; Subbarao et al., 1993; Ramírez and Dessen, 2000; Coluzzi et al., 2002). Moreover, deficits in heterozygotes and strong linkage disequilibrium among polymorphic rearrangements has provided evidence for the existence of reproductive isolation barriers and ongoing speciation processes (pre and post-zygotic) within species (Coluzzi, 1982; Costantini et al., 1999). Inversions have also been used as effective tools for phylogenetic analysis and chromosome evolution in the genus *Anopheles*. The study of rearrangements across species has made it possible to infer the phylogenetic tree in the *gambiae* complex (e.g., Xia et al., 2008; Sharakhova et al., 2011). In addition, the study of breakpoint architecture has led to elucidation of the ancestry of species and inversions in them (Sharakhov et al., 2006; White et al., 2009). Lately, inversions have also been shown to be key factors in ecological success across distinct A*nopheles* species. A clear example is the acquisition by *An. gambiae* of inversions *2La* and *2Rb* introgressed from *An. arabiensis* (Della Torre et al., 1997; White et al., 2009). The ancestral range of *An. gambiae* was the rainforest and acquisition of these inversions from an arid species is thought to have given it the ecological and genetic flexibility to invade the savannas, which has most likely contributed to the establishment of this species as the foremost malaria vector (Coluzzi et al., 1979; Della Torre et al., 1997; Besansky et al., 2003; Ayala and Coluzzi, 2005). Environmental and/or geographical clines have also linked inversions to local adaption, corroborated in some species by seasonal changes in inversion frequencies (Simard et al., 2009; Ayala et al., 2011a). However, there have been few phenotypic experiments, i.e., experiments to validate the functional relationship between candidate genes and the specific trait in question, and genetic experiments due to limitations imposed by the difficulty of breeding most of the *Anopheles* species under insectary conditions. Recently, renewed interest in inversions has been fueled by the availability and relatively low cost of genome sequencing technologies. These promising new tools are aimed at deciphering the origin and evolution of inversions and ultimately the genes directly linked to the phenotypic trait associated with the chromosomal rearrangement (Kirkpatrick and Kern, 2012).

Through an exhaustive literature survey, we review here the evidence for the adaptive role of chromosomal inversions in the genus *Anopheles*. A total of eight species and 49 inversions have been associated with different phenotypic traits in *Anopheles* species in various studies (Figure 1, Table 1), the majority of them on the basis of associations between inversion frequencies and environmental/behavioral traits in natural populations. More recently, genomics and transcriptomics approaches and phenotypic assays in insectary conditions have substantially increased our understanding of the adaptive role of *Anopheles* inversions. To our knowledge, this is the first review providing a general overview of patterns, traits, and species with regard to the role of inversions in *Anopheles* local adaptation.

**Table 1:**
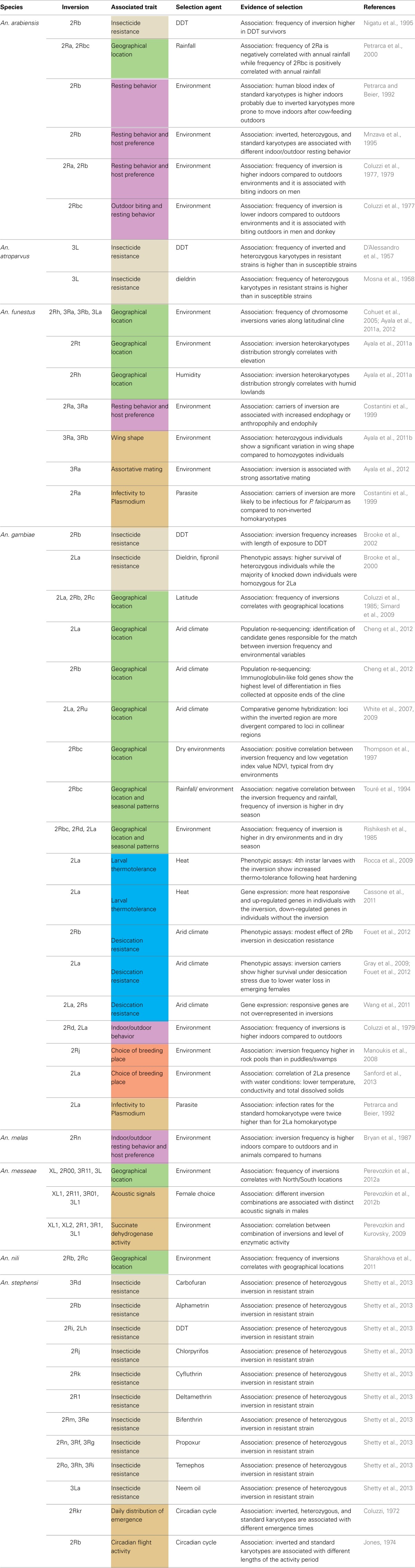
**Summary of traits, selection agents and evidence of selection available for 49 inversions described in eight different *Anopheles* species**.

## Associations between inversions, traits, and fitness

Inversions in *Anopheles* have been associated with several phenotypic traits, from insecticide resistance to behavioral characteristics and enzymatic activity (Table 1).

### Insecticide resistance

The first association studies linking chromosomal inversions with adaptation focused on the role of *Anopheles atroparvus* inversions in insecticide resistance (Figure 1) (D'Alessandro et al., 1957; Mosna et al., 1958). Since these initial studies, positive associations between inversions and different insecticides, both synthetic and natural, have been reported in three other species: *An. arabiensis, An. gambiae*, and *An. stephensi* (Figure 1, Table 1). Most research in this area is based on the study of inversion frequencies in artificially selected strains, although there are also some examples of associations between inversion frequencies and resistance in natural populations (Nigatu et al., 1995). Understanding the role of inversions in insecticide resistance could have direct implications for the success of malaria control programs by helping us understand the spread and introgression of resistance alleles between natural populations (Tripet et al., 2007; Enayati and Hemingway, 2010). One example of how inversions facilitate the spread of insecticide resistance alleles in mosquitos of the genus *Culex* is described by Labbé et al. (2007), who report that resistant alleles, which are highly deleterious when homozygous but adaptive when heterozygous, could potentially be kept in a heterozygous state through the presence of inversions.

### Environmental adaptation and geographical distribution

Frequency of chromosomal inversions has also been extensively associated with latitudinal, but less frequently with altitudinal, patterns in several *Anopheles* species (Table 1, Figure 1). In some cases, the particular environmental factor responsible for the association between the inversion and the clinal pattern has been identified (Touré et al., 1994; Petrarca et al., 2000). Polymorphic inversions non-randomly distributed both temporally and spatially have been reported in *An. gambiae*. More specifically, inversions are present at high frequencies or are fixed in xeric environments while they are virtually absent in mesic environments, and frequencies peak in dry seasons and trough in rainy seasons (Coluzzi et al., 1979). These patterns strongly suggest that inversions play a role in adaptation to xeric environments. Indeed, it has been shown that the presence of inversions can be predicted at 86% of the geographical sites studied on the basis of climatic variables, mean annual precipitation, evapotranspiration, minimum temperature, and maximum temperature having the greater explanatory power (Bayoh et al., 2001).

Several phenotypic experiments have been carried out to further characterize the selection agents responsible for spatial and temporal patterns of inversions in this malaria mosquito. Resistance to thermal and desiccation stresses were compared in carriers and non-carriers of inversion *2La* (Gray et al., 2009; Rocca et al., 2009; Fouet et al., 2012) and inverted chromosomes were indeed found to exhibit greater resistance to both stresses, as expected on the basis of their association with arid environments. Gray et al. (2009) further established that resistance to desiccation was due to lower rates of water loss and higher initial body water content in flies carrying the inversion, while Fouet et al. (2012) found the inversion itself to play only a weak role in body size and also showed a possible involvement of the inversion *2Rb* suggesting an epistatic effect of both inversions in desiccation resistance (Fouet et al., 2012).

### Behavioral traits

Aside from playing a role in insecticide resistance and the establishment and maintenance of clinal, altitudinal, and seasonal patterns, inversions have also been associated with behavioral characteristics such as indoor/outdoor resting and mate choice (Table 1).

Indoor resting behavior may be related to indoor environmental conditions as these generally display a higher nocturnal saturation deficit than the outdoor environment (Coluzzi et al., 1977). This hypothesis is supported in the cases of inversion *2Ra* in *An. arabiensis* and *2La* in *An. gambiae*, as the first is negatively associated with humidity and the second has been repeatedly associated with arid climates in independent studies (Table 1). Some of these studies also found correlations between the same inversions and host preference, i.e., human vs. cattle (Bryan et al., 1987; Costantini et al., 1999). Resting behavior has implications for vector control programs because mosquitos that exhibit a preference for resting inside houses are easier targets for current vector control programs (bednet distribution or indoor insecticide spray).

Inversions have also been associated with mating behavior in *An. funestus* (Figure 1), where it has been reported that between 77% and 91% of matings are assortative based on karyotype (Ayala et al., 2012). Partner selection behavior has consequences for fitness since it may suppress offspring maladapted to local conditions, for example by preventing the reduction in fitness caused when different alleles adaptive to different environments are brought together in a hybrid individual.

### Other associated traits

Evidence for associations between inversions and traits such as infectivity by *Plasmodium*, acoustic signals, breeding place, circadian cycle, and morphometric variation have also been reported.

Directly linked to malaria transmission, infectivity by *P. falciparum* has been associated with inversions in *An. funestus* and *An. gambiae* (Petrarca and Beier, 1992; Costantini et al., 1999). A cluster of resistance genes has been located within inversion *2La* in *An. gambiae* (Riehle et al., 2006). Although still controversial, several studies have revealed *Plasmodium* infection to incur a fitness cost to *Anopheles*, mainly in reduced fecundity and/or survival (Hurd et al., 2005).

In *An. messeae*, changes in acoustic signals have been observed across different inversion carriers (Figure 1, Table 1; Perevozkin et al., 2012b). Selection for this trait may modify mosquitoes' mating systems: changes in the reception of acoustic signals can prevent mating, leading to reduced gene flow between populations (Pennetier et al., 2010).

Other biological processes affecting mating in anophelines have also been described. In *An. stephensi*, the circadian cycle has been related to one specific inversion (Table 1) (Coluzzi, 1972; Jones, 1974). Mating behavior is directly driven by the internal circadian rhythms of *Anopheles* (Sawadogo et al., 2013a), which may easily be selected to prevent gene flow, for instance. Moreover, the circadian cycle affects biting behavior, and, therefore, potentially survival and dispersion (Rund et al., 2013).

Similarly, breeding place selection has been shown to be a trait directly linked to the inversion 2Rj in *An. gambiae* (Manoukis et al., 2008). Choice of breeding place has a direct impact on many critical aspects of the biology of mosquitoes, for instance, larva competition and density, which bring about a reduction in emergences, and hence in survival (Muriu et al., 2013). Moreover, the availability of suitable breeding sites determines the distribution of mosquito species (Mouchet et al., 2008).

Finally, morphometric variation has been associated with inversions in *An. funestus* and *An. gambiae* (Ayala et al., 2011b; Fouet et al., 2012). Body size has been found to be a significant factor in several biological characteristics in mosquitoes: host seeking, malaria transmission, mating, and fecundity (Takken et al., 1998; Russell et al., 2011; Yaro et al., 2012; Sawadogo et al., 2013b), which represent evolutionary selection targets under particular environmental conditions.

Taken together, these associations demonstrate the high diversity and complexity of traits associated with chromosomal inversions in *Anopheles* species. Classical studies, such as those we have just described, are a first necessary step toward understanding chromosomal inversions in adaptation. However, studies aimed at identifying the specific genes responsible for these associations are also needed in order to fully understand the role of chromosomal inversions in adaptation.

## Genomic and transcriptomic approaches to the study of inversions

Recently, the broad availability of genomic and transcriptomic techniques has allowed chromosomal inversions to be studied at an unprecedented level of detail. Although scarce, a few studies have been carried out aimed at identifying the candidate genes responsible for environmental adaptation in *An. gambiae*. A first step toward this goal was the use of comparative genome hybridization techniques to map divergent regions between chromosomal inversions (White et al., 2007, 2009). These studies have resulted in the discovery of two relatively small regions likely responsible for the maintenance of inversion *2La*, and one region likely responsible for inversion *2Ru*, while no divergent regions could be identified for the other chromosome 2 inversions. Follow-up analysis, using a population re-sequencing approach, resulted in identification of the candidate genes inside the *2La* and *2Ru* diverged regions (Cheng et al., 2012). Interestingly, some of the biological functions associated with environmental adaption in *An. gambiae*—such as gustatory receptors, ion-channel related genes and regulation of chromatin and transcription—are shared with *D. melanogaster* (Kolaczkowski et al., 2011). These results suggest that that there might be parallel adaptive responses to similar selective environmental pressures in different species, e.g., in adaptation to arid environments.

Thermal and desiccation stresses are common threats to insects living in arid environments. In an attempt to identify the specific genes involved in responses to these two stresses, microarrays were used to compare the genome-wide expression of genes before and after these stress conditions (Cassone et al., 2011; Wang et al., 2011). Cassone et al. (2011) found that a large number of genes were heat responsive and up-regulated in larvae carrying the *2La* inversion compared with larvae carrying the standard karyotype. Proteolytic, chaperone, and metabolic functions were over-represented in these genes (Cassone et al., 2011). On the other hand, Wang et al. (2011) did not find significant enrichment for genes involved in the response to desiccation in chromosomal inversions in *An. gambiae* mosquitos subject to acute desiccation stress. Given that both studies induced similar physiological stresses, we would expect both of them to identify candidate genes in the inverted regions. However, there were differences in the experimental conditions and in the mosquito developmental stages analyzed, which could explain, at least in part, the different results obtained in the two studies.

Capitalizing on the complete genome sequences and high-throughput molecular technologies already available, further studies should be devoted to understanding the genetic basis of *Anopheles* adaptation to local environmental conditions. To this end, inversions are compelling target genomic regions. Expression and genomic analyses should be linked to phenotypic assays to confirm the functional implications of the candidate genes identified. Eventually, all these analyses should pave the way to developing molecular assays to monitor the presence of inversions in the context of seasonal/environmental changes, which in turn will help in the design of more effective vector control strategies.

## Concluding remarks

The role of inversions in *Anopheles* adaptation has been the subject of study since the 1950s. Taken together, these studies reveal a variety of traits likely to be involved in adaptation. The classical explanation, proposed by Dobzhansky, is that inversions influence fitness by favoring the selection of co-adaptive alleles by reducing recombination (Dobzhansky, 1970). More recently, it has been shown that inversions containing locally adapted genes can spread in populations without the necessity of drift or coadaptation (Kirkpatrick and Barton, 2006). Alternatively, the genes flanking breakpoint regions may be altered when inversions occur (“position effects”; Sperlich, 1966), thus creating new structures (Mitelman et al., 2007) or modifying expression attributes (Pérez-Ortín et al., 2002; Puig et al., 2004), which may occasionally be selected and lead to adaptation. However, knowledge of the genes within inversions or affected by inversion breakpoints is still very limited. To date, the breakpoints of only three inversions in *An. gambiae*—2Rb (Lobo et al., 2010), 2Rj (Coulibaly et al., 2007), and 2La (Sharakhov et al., 2006)—and one in *A. arabiensis*—2Rd1 (Mathiopoulos et al., 1998)—have been characterized. The increasing availability of genomics and transcriptomics data from *Anopheles* will certainly accelerate identification of inversion genes likely playing a role in adaptation (Neafsey et al., 2013). Once identified, direct manipulation of these candidate genes to test the effects of specific alleles on a particular trait and in the same genetic background would provide direct evidence for their causal effect. Because of the limitations in carrying out experiments with *Anopheles* species in the laboratory, *D. melanogaster* may be used as a model to assess the effects of some of these candidate genes, as has already been done with insecticide resistance genes (Daborn et al., 2012). In addition, the availability of whole genome sequences will facilitate analysis of other types of genomic variants that may also be affecting the ability of *Anopheles* to adapt to different environments (Casacuberta and González, 2013; Neafsey et al., 2013).

## Author contributions

Diego Ayala, Anna Ullastres and Josefa González contributed to the drafting and writing of this review.

### Conflict of interest statement

The authors declare that the research was conducted in the absence of any commercial or financial relationships that could be construed as a potential conflict of interest.
